# Identification of a novel and a recurrent *CDC45* variant in a Chinese family with Meier-Gorlin syndrome 7 and a literature review

**DOI:** 10.3389/fgene.2026.1893476

**Published:** 2026-08-24

**Authors:** Ying Zhao, Yiyang Fu, Shuying Zhang, Yuxin Han, Jinhui Zhu, Wanqi Liu, Shuxian Li, Dan Xiao, Yuan Liu, Lin Wang, Ruixue Zhang, Wenjing Cheng, Kaitao Ren, Xiaorui Zhang, Mingyue Chen, Abdullah, Umm-e- Kalsoom, Nousheen Bibi, Saadullah Khan, Fengni Fan, Ting Jia, Xiangdong Lin, Wei Li, Zhao Li, Gen Nishimura, Takahiro Yamada, Na Cai, Chisa Shukunami, Zhiqiang Tian, Xiaoxu Chen, Shiro Ikegawa, Rong Qiang, Long Guo

**Affiliations:** 1 Department of Pediatric Endocrinology and Metabolism, Northwest Women’s and Children’s Hospital, The Affiliated Northwest Women’s and Children’s Hospital of Xi’an Jiaotong University, Northwest Women’s and Children’s Hospital, Xi’an, China; 2 Department of Laboratory Animal Science, School of Basic Medical Sciences, Xi’an Jiaotong University Health Science Center, Xi’an, China; 3 Center of Medical Genetics, Northwest Women’s and Children’s Hospital, The Affiliated Northwest Women’s and Children’s Hospital of Xi’an Jiaotong University Health Science Center, Northwest Women’s and Children’s Hospital, Xi’an, China; 4 Department of Biochemistry, Hazara University, Mansehra, Pakistan; 5 Department of Bioinformatics, Shaheed Benazir Bhutto Women University, Peshawar, Pakistan; 6 Department of Biotechnology and Genetic Engineering, Kohat University of Science & Technology (KUST), Kohat, Pakistan; 7 Department of Radiology, Musashino-Yowakai Hospital, Tokyo, Japan; 8 Division of Clinical Genetics, Hokkaido University Hospital, Sapporo, Japan; 9 Department of Molecular Biology and Biochemistry, Graduate School of Biomedical and Health Sciences, Hiroshima University, Hiroshima, Japan; 10 School of Software Engineering, Xi’an Jiaotong University, Xi’an, China; 11 College of Animal Science and Technology, Northwest A&F University, Yangling, Shaanxi, China; 12 Laboratory for Statistical and Translational Genetics, RIKEN Center for Integrative Medical Sciences, Yokohama, Japan

**Keywords:** *CDC45*, growth, growth hormone, Meier-Gorlin syndrome, short stature

## Abstract

**Introduction:**

Meier-Gorlin syndrome 7 (MGORS7) is a rare autosomal recessive disorder characterized by primordial dwarfism, craniosynostosis, and patellar aplasia, caused by pathogenic variants of *CDC45*. Here, we report a Chinese patient presenting with classic hallmarks of MGORS7 alongside atypical clinical features, including hearing and visual impairments.

**Methods:**

Clinical and radiological data were collected. Whole-genome sequencing and Sanger sequencing were performed to identify and validate the causative variants. Their functional effects were investigated using an exon-trapping assay, and a literature review of previously reported MGORS7 cases was conducted.

**Results:**

Genetic analysis identified two compound heterozygous *CDC45* variants: c.1416C>T (p.H472=) and c.1559+2T>A, which are a recurrent variant in the East Asian population and a novel variant, respectively. Our exon-trapping assay indicated that c.1559+2T>A induced aberrant splicing, generating transcripts predicted to undergo nonsense-mediated mRNA decay. Additionally, growth hormone therapy was initiated in our patient, with a noted improvement in growth parameters in the initial assessment and without immediate complications. The literature review identified a total of 32 *CDC45* variants in 29 patients with MGORS7, who showed high heterogeneity in clinical phenotypes.

**Discussion:**

Our study further expanded the mutational spectrum of *CDC45* and provided a preliminary clinical observation suggesting that growth hormone therapy may be beneficial for growth retardation in patients with MGORS7.

## Introduction

Meier-Gorlin syndrome (MGORS) is a rare genetic disease presenting with microcephalic primordial dwarfism, which was first proposed by Meier in 1959 (Meier et al., 1959). Gorlin further described MGORS with similar phenotypes in 1975 (Gorlin et al., 1975). Characteristic features of MGORS include intrauterine growth retardation, postnatal short stature, microtia, patella hypoplasia/aplasia, abnormal genitalia, and musculoskeletal abnormalities (de Munnik et al., 2015).

MGORS is causally associated with 13 genes, including *ORC1, ORC4, ORC6, CDT1, CDC6, GMNN, MCM3, MCM5, MCM7, CDC45, GINS2, GINS3,* and *DONSON* (Nielsen-Dandoroff et al., 2023; Kingsley et al., 2023). Most MGORS subtypes are inherited in an autosomal recessive manner, whereas MGORS6, caused by heterozygous pathogenic variants in *GMNN*, follows an autosomal dominant inheritance pattern. All these genes play essential roles in the early DNA replication processes. Therefore, MGORS is regarded as a syndrome resulting from disrupted DNA replication. Among these 13 genes, biallelic pathogenic variants in *CDC4*5 specifically cause Meier-Gorlin syndrome 7 (MGORS7, OMIM: # 617063), one distinct subtype within the MGORS spectrum. In addition to the shared clinical features of MGORS, such as short stature, microtia, and patellar hypoplasia, MGORS7 is distinguished by its characteristic presentation of craniosynostosis (Fenwick et al., 2016).

*CDC45* encodes cell division control protein 45, a critical regulator of the cell cycle. *CDC45* activates the latent MCM2-7 helicase by binding GINS hetero-tetramer to form CDC45/MCM2-7/GINS (CMG) complex, which unwinds the DNA duplex and initiates replication (Petojevic et al., 2015). In addition, CDC45 and GINS stabilize the spatial architecture of the MCM2-7 complex and secure the leading-strand DNA within the central channel, preventing the single-stranded DNA from slipping out under replication-stress conditions (Denkiewicz-Kruk et al., 2025). While altered expression of CDC45 is associated with the progression of multiple cancers (Sun et al., 2017; Huang et al., 2019; Yang et al., 2020), dysfunction of both alleles of *CDC45* results in MGORS7.

In this study, we described a Chinese boy with MGORS7, who was a compound heterozygote for *CDC45* variants*,* c.1416C>T and c.1559 + 2T>A. c.1416C>T is a known recurrent pathogenic variant that could cause exon skipping (Schoch et al., 2024). c.1559 + 2T>A is a novel variant, which was demonstrated to be pathogenic by us using an exon trapping assay. Additionally, our patient received treatment with long-acting pegylated recombinant human growth hormone (PEG-rhGH), which was associated with improved growth velocity.

## Materials and methods

### Patient

A 7-year-old Chinese boy was referred to us because of postnatal growth retardation. He is the first child of healthy, non-consanguineous parents with no family history of hereditary disorders. Prenatal ultrasonography indicated intrauterine growth restriction. The patient was born at full term with low birth weight (2.2 kg, −2.3 SD), short length (46 cm, −2.5 SD), and an occipitofrontal circumference of 34 cm. At 2 months of age, the patient was diagnosed with bilateral coronal craniosynostosis and subsequently underwent comprehensive craniofacial reconstruction surgery. At 11 months of age, the patient was found to have wide subdural space in the left frontotemporal region and a pointed downward displacement of the cerebellar tonsils with mild herniation into the spinal canal, which necessitated the second craniomaxillofacial reconstruction. Besides, he presented with bilateral strabismus, refractive error, and amblyopia in the right eye, for which he underwent strabismus correction surgery combined with inferior fornixplasty at the age of 5.5 years.

**FIGURE 1:**
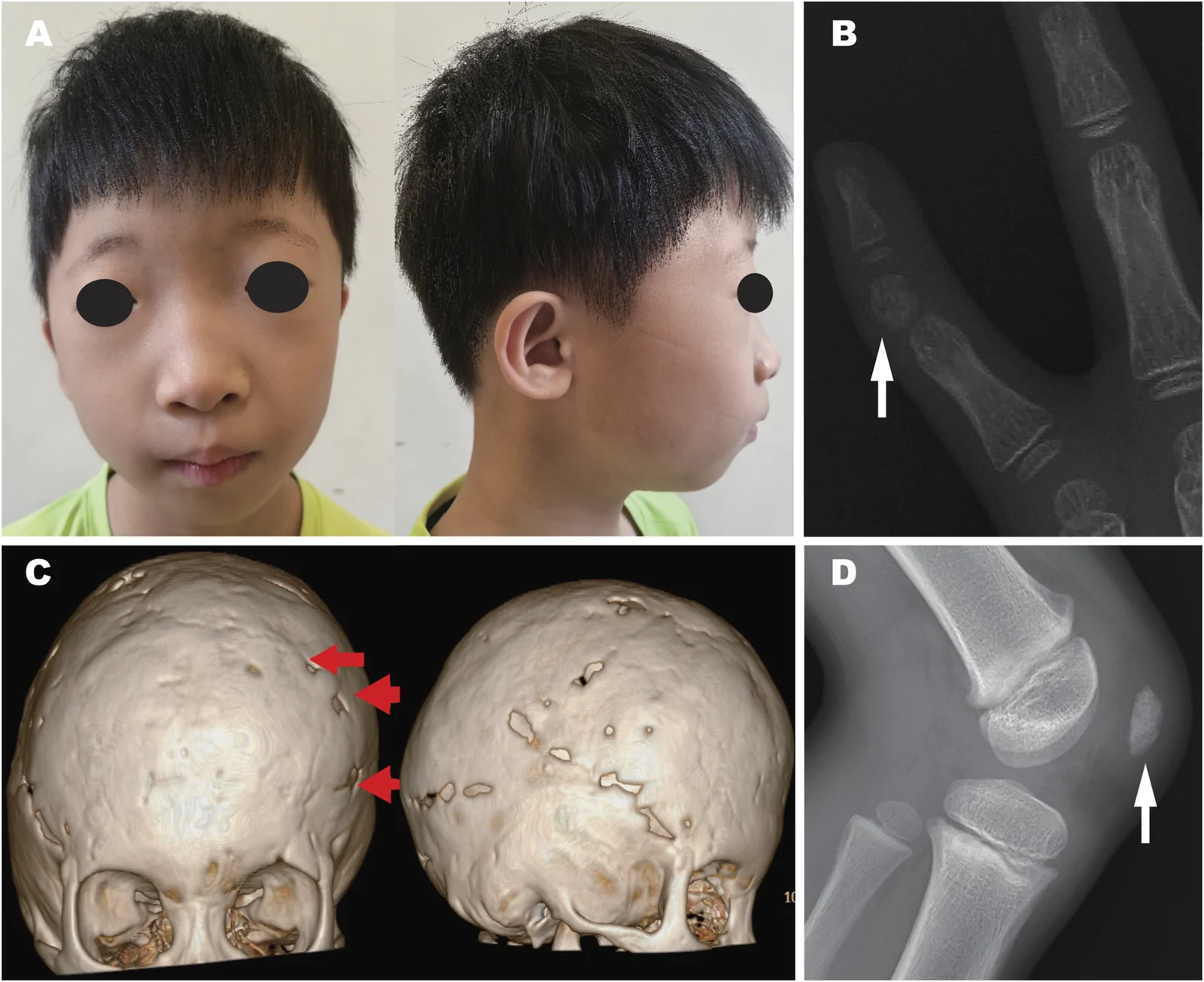
Skeletal abnormalities of the patient. **(A)** Craniofacial features. Supraorbital depression, minor mouth corner deviation, microstomia, prominent lips, and retrognathia. **(B)** Shortening of the left fifth middle phalanx (white arrows). **(C)** Cranial CT with VR reconstruction showing irregular skull contour with a widened forehead and elongated anteroposterior diameter. Multiple cranial sutures (frontal, sagittal, coronal, and lambdoid) and fontanelles are closed. Red arrows indicate multiple areas of irregular bony defects. **(D)** Patellar hypoplasia (white arrow).

The physical examination at 7.3 years showed a weight of 15 kg (−2.8 SD), a height of 116 cm (−2 SD) and a head circumference of 46 cm (−2.5 SD). Dysmorphic features included hypertelorism, bilateral supraorbital ridge hypoplasia, slight asymmetry of the labial commissures, microstomia, retrognathia, a high-arched palate, and dental malalignment (Figure 1A). In addition, he exhibited bilateral brachydactyly and clinodactyly of the 5th fingers, with irregular morphology noted in the phalanges of the left 5th digit (Figure 1B). His bone age was assessed to be 2 years based on carpal radiographs, demonstrating a significant delay in skeletal development. Skeletal survey showed multiple skull bone defects and abnormal cranial contour. The sagittal, coronal and lambdoid sutures, as well as the anterior and posterior fontanelles, were already closed, indicating premature cranial suture closure (craniosynostosis) (Figure 1C). Bilateral hypoplastic patellae were noted (Figure 1D), while the hip joints and spine were normal. He had conductive hearing loss in the left ear. Insulin-like growth factor 1 (IGF-1) was 173 ng/mL (0 SD), while growth hormone (GH) peak value was 16 ng/mL with an arginine-levodopa combined stimulation test. No abnormality was found in the cardiac, hepatobiliary, pancreatic, splenic, and urinary tract ultrasonography.

### Whole-genome sequencing (WGS)

**FIGURE 2:**
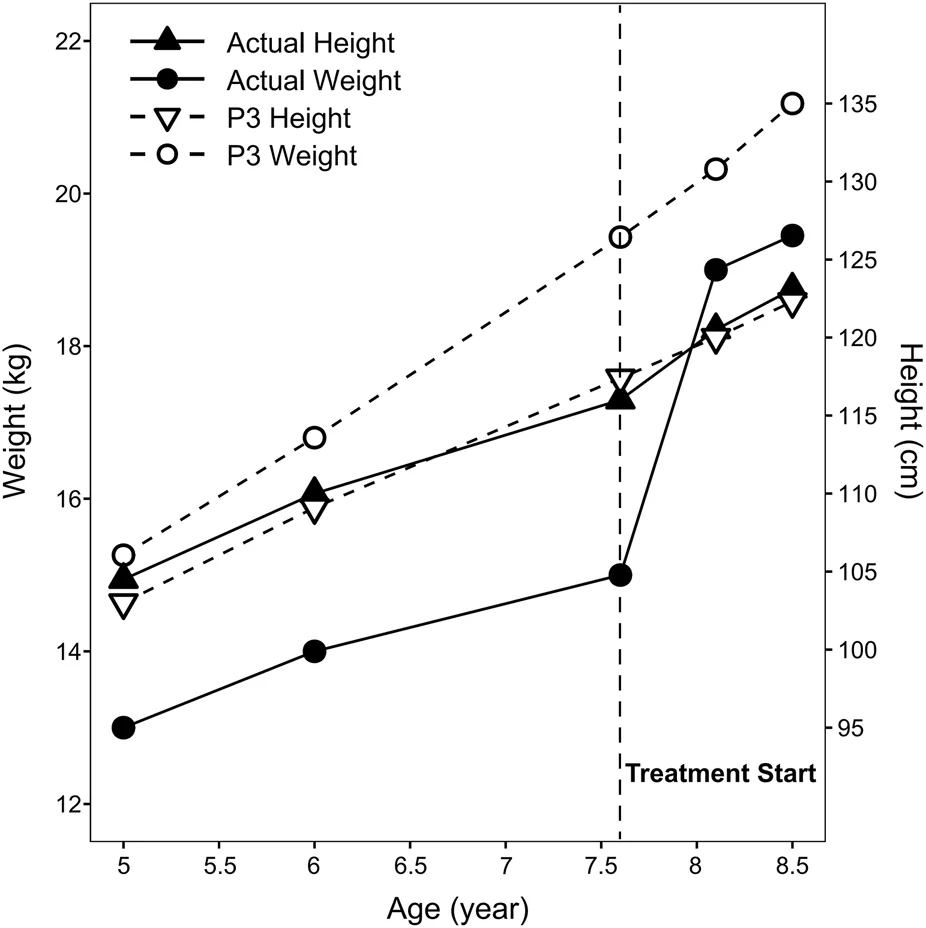
Response to growth hormone (GH) treatment. Height and weight measurements of the patient before and after the initiation of GH treatment (starting at 7.6 years of age). The P3 curve represents the 3rd percentile of age- and sex-matched reference values based on Chinese growth standards.

**FIGURE 3:**
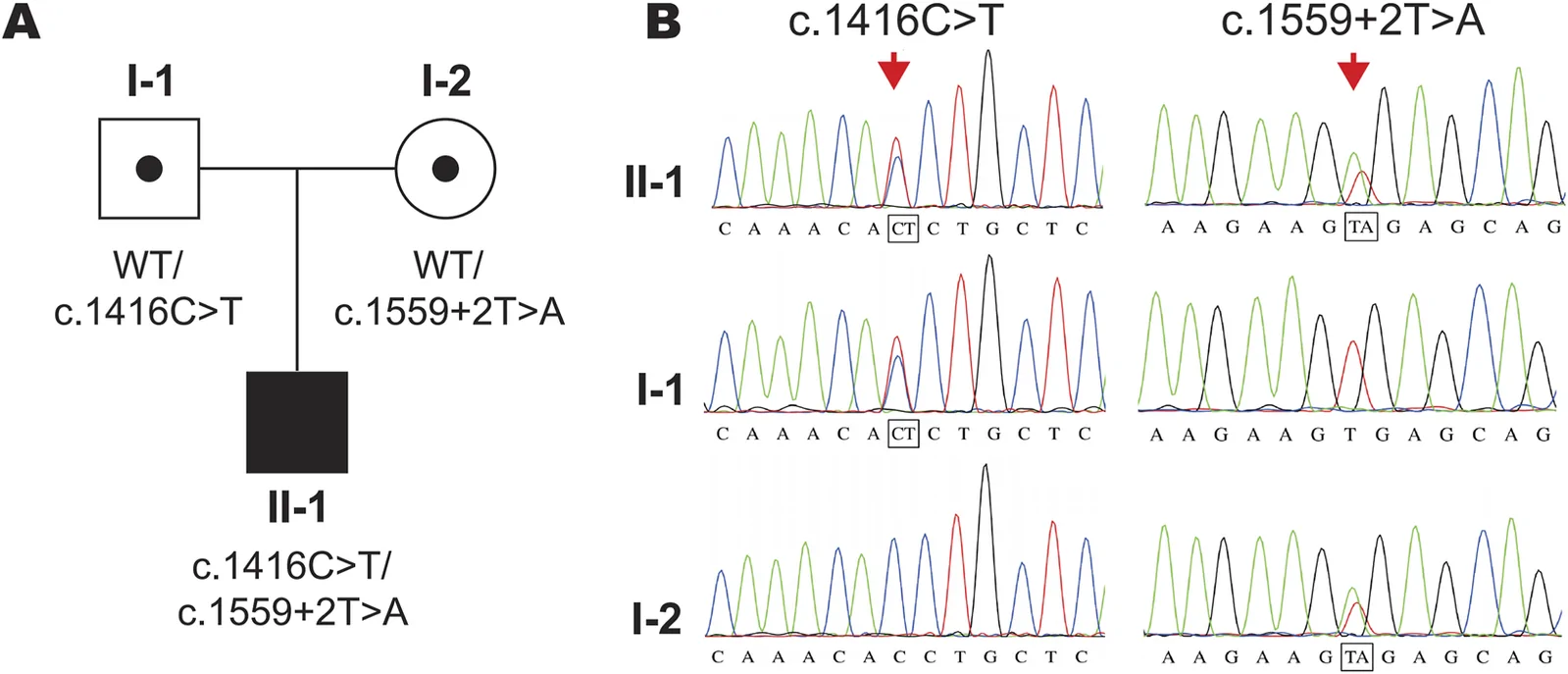
Identification of CDC45 variants in the patient and the parents. **(A)** Pedigree of the family. The proband (II-1) is a compound heterozygote. The father (I-1) and mother (I-2) are asymptomatic carriers. **(B)** Sanger sequencing chromatograms confirming the compound heterozygous state of the patients.

This study conformed to the declaration of Helsinki and was approved by the Ethics Committee of Northwest Women’s and Children’s Hospital (No. 21–036). Peripheral blood was collected from the proband and his parents (I-1, I-2, II-1 in Figures 2A, 3A). Informed consents were obtained from the family. Trio WGS was performed on the patient and his parents using the Illumina high-throughput sequencing platform. Genomic DNA was extracted from peripheral blood and subjected to quality control, followed by library preparation using the NEB Next® Ultra™ DNA Library Prep Kit for Illumina (NEB, USA). After fragmentation and index ligation, the libraries were clustered on the cBot Cluster Generation System and subsequently sequenced on an Illumina platform, generating 150 bp paired-end reads. Valid sequencing data is mapped to the human reference genome GRCh38 using Burrows Wheeler Aligner (BWA) (Li and Durbin, 2010). The resulting BAM files were sorted with SAMtools (Danecek et al., 2021) and Sambamba (Tarasov et al., 2015). Variant calling was performed using SAMtools, Control-FREEC (Boeva et al., 2011), and LUMPY (Layer et al., 2014), and variant annotation was carried out with ANNOVAR (Wang et al., 2010). Variants were subsequently filtered based on population allele frequency, predicted functional impact, consistency with the expected inheritance pattern, and the patient’s clinical phenotype. Candidate variants were then selected for Sanger sequencing validation and further pathogenicity evaluation. Detailed sequencing quality metrics, including sequencing depth and genome coverage, are provided in the Results section and summarized in Supplementary Table S1.

### Sanger sequencing

Sanger sequencing was performed on a 3500 Dx DNA analyzer (Applied Biosystems) to confirm the variants identified by WGS. Fragments including the variants were amplified by PCR using primers, 5′-ACA​GAG​CAC​AGG​CGC​CTG​GTC-3′ and 5′-GCT​GCT​GAC​ACA​CAA​GAC​ATG​TCG-3′. Sequencher V.4.7 (Gene Codes) was used for aligning sequencing chromatographs to reference sequences.

### Variant evaluation

Variant interpretation was performed according to the ACMG/AMP guidelines using information from dbSNP (https://www.ncbi.nlm.nih.gov/snp/), ClinVar (https://www.ncbi.nlm.nih.gov/clinvar/), Genome Aggregation Database (gnomAD: https://gnomad.broadinstitute.org/), and the Westlake BioBank for Chinese (WBBC: https://wbbc.westlake.edu.cn/). The pathogenicity of the variant located at the exon-intron junction was evaluated using online splice-site evaluation tools, including SpliceAI (https://spliceailookup.broadinstitute.org/) and RDDC (https://rddc.tsinghua-gd.org/zh/tool/rna-splicer).

A literature search was performed in PubMed, OMIM, and the Human Gene Mutation Database (HGMD) using the keywords “CDC45”and “Meier-Gorlin syndrome 7”. Published cases with genetically confirmed *CDC45* variants and clearly documented clinical phenotypes were included. Clinical and genetic data were extracted from the eligible reports for descriptive analysis.

### Cell culture

HEK293T cells were cultured in DMEM medium (Gibco, USA) supplemented with 10% fetal bovine serum (Gibco) and 1% penicillin/streptomycin (Coolaber, China) at 37 °C in a humidified atmosphere with 5% CO2. Cells were seeded into 24-well plates for an exon trapping assay and transfected with Lipofectamine™ 2000 Reagent (Invitrogen, USA).

### Exon trapping assay

Exons 16–18 of *CDC45* were PCR amplified from control genomic DNA using a forward primer located in intron 15 (5′-TCG​CTC​GAG​AGT​GGA​GTG​ACT​GGG​CGC​TGT​T-3′) and a reverse primer located in intron 18 (5′-CGG​GAT​CCT​CAA​GAA​CCC​TGC​TTC​GGT​CTG-3′). PCR products were digested with *Xho*I and *Bam*HI, and cloned into an exon trapping vector (Mo Bi Tec, Germany) as a wild type plasmid. Site-directed mutagenesis of c.1559 + 2T>A was performed using KOD-Plus-Mutagenesis Kit (Toyobo, Japan) by reverse PCR amplification, with forward primer: 5′-GAG​AGC​AGC​TTC​CAC​TCG​TCC​T-3′ and reverse primer: 5′-TTC​TTC​CTG​TCC​GAG​CTG​TCG​GT-3’. Exon trapping vectors were transfected into HEK293T cells. After 24 h, total RNA was extracted (Invitrogen) and reverse-transcribed to obtain cDNA (RevertAid kit). RT-PCR was performed using primer sets supplied with the exon trapping assay kit and PCR products were sequenced.

## Results

### GH treatment and its initial assessment

In light of the patient’s severe growth retardation, with both height and weight significantly below those of age- and sex-matched peers, the patient was initially diagnosed with idiopathic short stature (ISS) based on the clinical phenotype. GH therapy was initiated in accordance with the ISS guidelines (Allen, 2011; Danowitz and Grimberg, 2022; Frindik and Kemp, 2010). The patient received PEG-rhGH at a dose of 0.2 mg/kg/week. After 10 months of treatment, the patient’s height increased by 7.1 cm and weight by 4.45 kg, representing a marked acceleration in growth velocity from 3.75 cm/year to 7.89 cm/year and weight gain from 0.625 kg/year to 4.94 kg/year as compared to the period prior to medication (Figure 2; Table 1). Correspondingly, height improved from −2.0 SD to approximately −1.7 SD, and weight improved from −2.8 SD to approximately −2.5 SD.

**TABLE 1:** Growth velocity before and after growth hormone therapy.

Period (age)	Actual height growth rate (cm/year)	Actual weight growth rate (kg/year)	P3 height growth rate (cm/year)	P3 weight growth rate (kg/year)
6–7.6(before therapy)	3.75	0.625	4.625	1.64
7.6–8.5(after therapy)	7.89	4.94	5.44	1.94

### Identification of biallelic *CDC45* variants

To achieve a more accurate molecular diagnosis, we performed whole-genome sequencing (WGS) on the patient and his parents. Clean sequencing data of 139.1 Gb, 145.4 Gb, and 172.8 Gb were obtained for the patient, father, and mother, respectively. The mean sequencing depth reached at least 44.2 reads, and at least 93.57% of the genomic regions achieved 20 reads coverage (Supplementary Table S1). We identified two variants in *CDC45* in the patient (Figure 3A). c.1416C>T was inherited paternally and c.1559 + 2T>A, maternally. The co-segregation of the two variants in the family was confirmed by Sanger sequencing (Figure 3B).

### *In silico* analysis of *CDC45* variants

**FIGURE 4:**
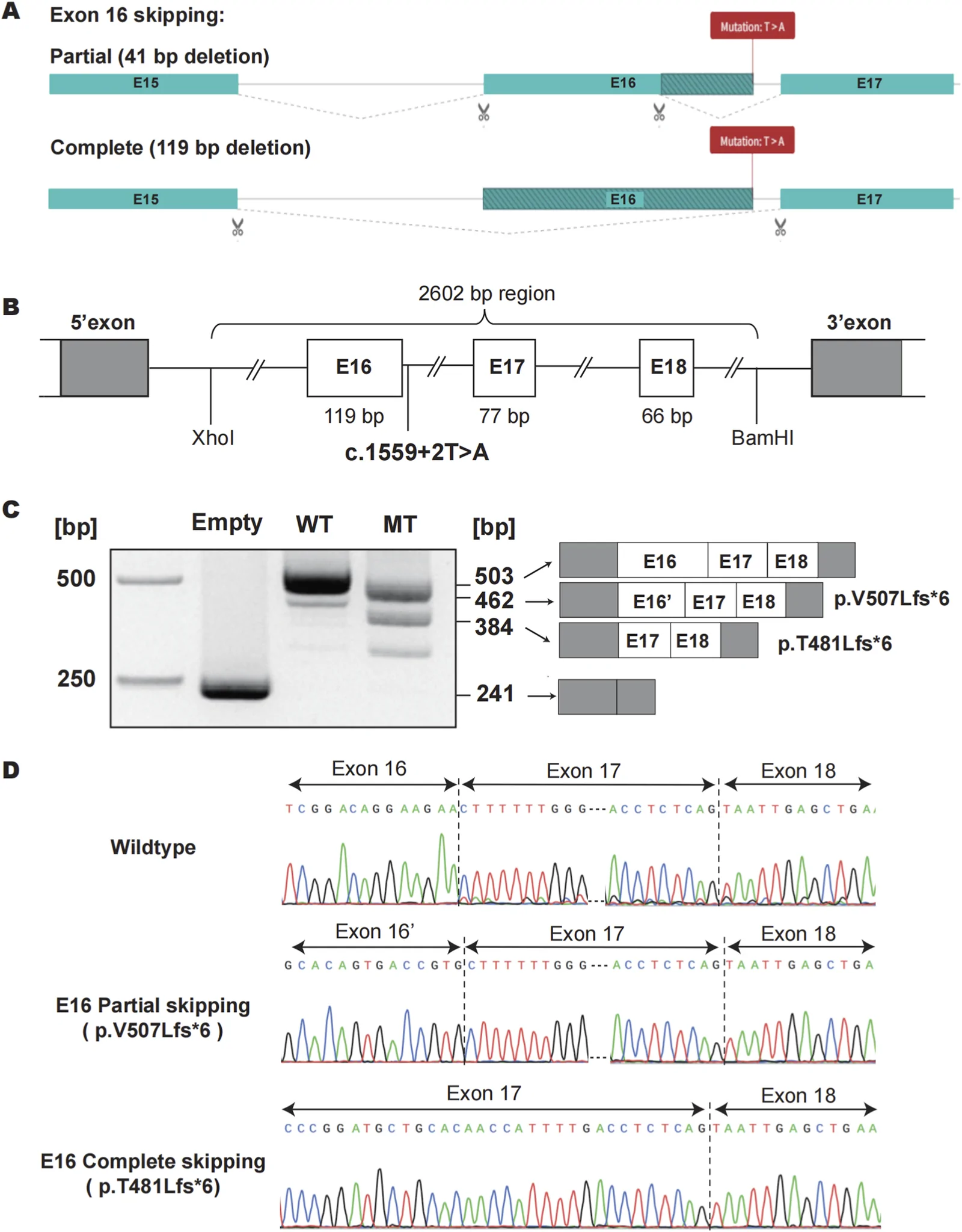
c.1559+2T>A causes aberrant splicing due to skipping of exon 16. **(A)** RDDC RNA Splicer predicted that the variant would produce two aberrant transcripts due to complete and partial skipping of exon(E)16. The former resulted in a 119-bp deletion and the latter a 41-bp deletion. **(B)** Schematic diagram of the exon trapping plasmids. **(C)** Exon trapping assay using HEK-293T cells. The mutant construct produced two aberrant splicing products as predicted: one containg a complete deletion of exon 16 and the other containing a partial deletion of 3′ of exon 16 (E16′). **(D)** Sanger sequencing of the RT-PCR products.

Schoch et al. (2024) have demonstrated that c.1416C>T was recurrent in two MGORS7 families. Using the exon-trapping assay similar to us, they showed that c.1416C>T could induce exon 15 skipping, thus leading to an in-frame deletion of 28 amino acids of CDC45. c.1559 + 2T>A was not deposited in the gnomAD and WBBC databases. *In silico* prediction using SpliceAI and RDDC indicated a high probability of aberrant splicing. Two patterns of splicing were predicted (Figure 4A).

### Functional verification of c.1559 + 2T>A

To validate the functional effect of c.1559 + 2T>A on the splicing, we performed an exon trapping assay (Figure 4B). The mutant construct displayed aberrant splicing, generating two abnormal transcripts. One transcript arose from activation of a cryptic splice donor site in exon 16, leading to a 41-bp partial deletion of 3′ side of exon 16. The other resulted from skipping of entire exon 16, producing a transcript with a 119-bp deletion (Figures 4C,D). Both aberrantly spliced transcripts contained premature termination codons (p.T481Lfs*6 and p.V507Lfs*6) and are predicted to be targeted for nonsense-mediated mRNA decay, thereby resulting in loss of function.

Based on the ACMG/AMP guidelines, the novel splice-site variant c.1559 + 2T>A fulfilled the criteria of PVS1 (a canonical splice-site variant demonstrated to cause aberrant splicing and predicted to result in loss of function), PM3 (detected in trans with the pathogenic c.1416C>T variant in a patient with an autosomal recessive disorder), and PM2_Supporting (absent from population databases). Therefore, the variant was classified as pathogenic.

### Mutational spectrum of *CDC45* in MGORS7

To date, including this case, 32 *CDC45* variants have been reported in 29 patients from 23 unrelated families with MGORS7 (Table 2). Among these, five patients carrying a 22q11.2 chromosomal deletion in trans with a pathogenic variant in the remaining *CDC45* allele have also been described. Of the 32 reported variants, missense variants were the most common, accounting for 15 variants. In addition, five splice-site variants, four synonymous variants, three frameshift variants, and one nonsense variant were demonstrated or predicted by functional assays or *in silico* analyses to alter the *CDC45* reading frame.

**TABLE 2:** Summary of *CDC45* variants in patients with MGORS7.

Variant	Location	Variant type	References
c.1A>C	exon 1	Initiation codon variant	Fenwick et al. (2016)
c.203A>G	exon 3	Missense variant	Fenwick et al. (2016)
c.204G>A	exon 3	Synonymous variant	Schoch et al. (2024)
c.226A>C	exon 4	Missense variant	Fenwick et al. (2016)
c.241G>A	exon 4	Missense variant	Unolt et al. (2020)
c.290T>C	exon 4	Missense variant	Schoch et al. (2024)
c.318C>T	exon 4	Synonymous variant	Fenwick et al. (2016)
c.333C>T	exon 4	Synonymous variant	Fenwick et al. (2016)
c.326_329dupTATA	exon 4	Frameshift variant	Li et al. (2021), Ting et al. (2020)
c.464A>G	exon 5	Missense variant	Fenwick et al. (2016)
c.469C>T	exon 5	Missense variant	Fenwick et al. (2016)
c.542 + 130 C>G	intron 6	Deep intronic variant	Unolt et al. (2020)
c.542 + 212G>A	intron 6	Deep intronic variant	Knapp et al. (2021)
c.653 + 5G>A	intron 8	Splice-site variant	Fenwick et al. (2016)
c.677A>G	exon 9	Missense variant	Fenwick et al. (2016)
c.751C>T	exon 10	Missense variant	Unolt et al. (2020)
c.791C>A	exon 10	Missense variant	Fenwick et al. (2016)
c.893C>T	exon 11	Missense variant	Fenwick et al. (2016)
c.961C>A	exon 12	Missense variant	Fenwick et al. (2016)
c.1021C>T	exon 13	Missense variant	Li et al. (2021)
c.1270C>T	exon 14	Nonsense variant	Fenwick et al. (2016)
c.1388C>T	exon 15	Missense variant	Fenwick et al. (2016)
c.1416C>T	exon 15	Synonymous variant	This study; Schoch et al. (2024), Ting et al. (2020)
c.1440 + 1G>A	intron 15	Splice-site variant	Schoch et al. (2024)
c.1440 + 14C>T	intron 15	Splice-site variant	Fenwick et al. (2016)
c.1445_1448del	exon 16	Frameshift variant	Knapp et al. (2021)
c.1487C>T	exon 16	Missense variant	Fenwick et al. (2016)
c.1532delC	exon 16	Frameshift variant	Fenwick et al. (2016)
c.1559 + 2T>A	intron 16	Splice-site variant	This study
c.1660C>T	exon 18	Missense variant	Fenwick et al. (2016)
c.*1 + 5G>A	intron 18	Splice-site variant	Schoch et al. (2024)
c.*36G>C	exon 19	3′UTR variant	Unolt et al. (2020)

Variant locations are annotated according to transcript NM_003504.4.

## Discussion

By systematically reviewing the clinical features of all MGORS7 patients, we observed that short stature, low weight, microcephaly, absent/hypoplastic patellae and craniosynostosis are present in nearly all affected individuals (Table 3). Moreover, patients with 22q11.2 deletions tend to exhibit more frequent and severe congenital heart defects compared with those carrying biallelic *CDC45* variants (Fenwick et al., 2016; Schoch et al., 2024; Li et al., 2021; Knapp et al., 2021; Ting et al., 2020; Unolt et al., 2020). At present, the limited number of reported cases precludes definitive conclusions regarding genotype–phenotype correlations among patients with biallelic *CDC45* variants. Nevertheless, the substantial clinical variability reported to date suggests that factors beyond the *CDC45* genotype may influence disease severity and phenotypic expression. Beyond these hallmarks, our patient presented with additional manifestations, including phalangeal deformity, hearing impairment, and visual disturbances. These deficits rarely occur across the reported cases (Table 3). Besides, other features associated with the MGORS7 spectrum, such as microtia, genital abnormalities, anorectal malformations, cardiac defects, and scoliosis, were not observed in our case. This phenotypic divergence further underscores the significant genetic heterogeneity and clinical complexity inherent in MGORS7.

**TABLE 3:** Clinical features of patients with Meier-Gorlin syndrome seven.

Clinical features	This study	Previously reported study					
		Biallelic *CDC45* variants (n = 24)^a^			22q11.2 heterozygous deletion + Monoallelic *CDC45* variant (n = 5)^b^		
		+	–	NA	+	–	NA
Clinical finding							
Short stature	+	17	5	2	2	3	0
Low body weight	+	18	4	2	2	1	2
Microcephaly	+	22	1	1	1	2	2
Microtia	–	21	3	0	2	1	2
Thin eyebrow	–	19	1	4	0	0	5
High palate	+	8	15	1	0	0	5
Finger/Toe deformity	+	9	15	0	4	1	0
Genital anomaly	–	6	13	5	2	3	0
Urinary system anomaly	–	5	15	4	1	3	1
Ano-rectal malformation	–	11	11	2	3	0	2
Radiogic finding							
Craniosynostosis	+	22	2	0	3	2	0
Absent/small patella	+	9	5	10	1	2	2
Cardiac defect	–	11	13	0	5	0	0
Vertebral column abnormality	–	4	19	1	2	1	2
Joint dislocation/laxity	–	6	12	6	1	2	2
Other abnormality							
Ophthalmological	Amblyopia, Strabismus	5	13	6	0	0	5
Auditory	Left ear hearing loss	4	13	7	2	0	3
Treatment	Surgury, PEG-rhGH	Surgury (2), Lucrin depot injection (1)	0	22	Surgury (5)	0	0

NA, not available; PEG-rhGH, long-acting pegylated recombinant human growth hormone. (): number of patients received the treatment.

^a^ Schoch et al. (2024), Fenwick et al. (2016), Ting et al. (Frindik and Kemp, 2010), Knapp et al. (Danowitz and Grimberg, 2022), Li et al. (Allen, 2011).

^b^ Unolt et al. (Li et al., 2021).

Among previously reported patients with MGORS7, seven have undergone clinical interventions. Most of these were surgical procedures, including craniofacial reconstruction and cardiac repair (Table 3). To date, pharmacological interventions targeting growth have been rarely reported. One patient was treated with a gonadotropin-releasing hormone (GnRH) agonist (Lucrin Depot) in an attempt to prolong the growth period; however, no significant effect was observed (Ting et al., 2020).

Before a molecular diagnosis was established, our patient fulfilled the clinical criteria for idiopathic short stature (ISS). Therefore, the initiation of PEG-rhGH was consistent with current international guidelines for the management of ISS (Allen, 2011; Danowitz and Grimberg, 2022; Frindik and Kemp, 2010). In clinical practice, GH therapy has traditionally been reserved for patients with documented GH deficiency, and children with normal IGF-1 levels are generally considered less likely to benefit from treatment (Vakili et al., 2022; de Munnik et al., 2012). However, this paradigm was challenged by Li et al. (2024), who reported a patient with MGORS4 and normal GH and IGF-1 levels who showed improved growth after 5 years of PEG-rhGH therapy. Similarly, our patient with MGORS7 also presented with normal baseline GH and IGF-1 levels, yet showed an encouraging increase in growth velocity during the initial assessment. After 10 months of treatment, the patient’s height exceeded the 3rd percentile for age- and sex-matched healthy cohorts. Although body weight remained below the standard range, it showed a substantial increase. Interestingly, during the first 6 months of rhGH therapy, the patient exhibited a more rapid increase in body weight compared with that observed during months 6–10 of treatment, whereas the height growth velocity remained relatively stable between the two periods. The marked early increase in body weight may reflect an initial catch-up anabolic response, including increased lean body mass accumulation and metabolic recovery, a phenomenon that has also been reported in children born small for gestational age receiving GH therapy. The subsequent slowing of weight gain may indicate a transition from the initial accelerated catch-up phase to a more stable maintenance growth phase (Xie et al., 2024; Gregory et al., 1991; De Schepper et al., 2008).

Although our 10-month anthropometric data suggest that PEG-rhGH therapy holds therapeutic potential for mitigating growth retardation in MGORS7, certain limitations must be acknowledged. First, serial serum IGF-1 measurements were not performed during the follow-up period, which limits our ability to evaluate the underlying biochemical response and correlate it with the observed clinical growth acceleration. Longitudinal follow-up is imperative to continuously track bone age maturation and monitor long-term safety profiles, ensuring that the accelerated growth velocity does not compromise final adult height. Furthermore, given the single-case nature of this report, these findings cannot be generalized to all affected individuals. To address this, we aim to establish an expanded cohort of MGORS7 patients. Future research with larger sample sizes will be critical to comprehensively evaluate the indications, efficacy, and safety of growth hormone interventions, potentially establishing a standardized therapeutic strategy for growth failure within the MGORS7 spectrum.

## Conclusion

In conclusion, we identified a novel splice-site variant and a recurrent synonymous variant in *CDC45* in a Chinese patient with MGORS7 and demonstrated the pathogenic effect of the novel variant through functional analysis. Our findings further expand the mutational and phenotypic spectrum of MGORS7 and provide additional insights into the genotype–phenotype correlation of *CDC45*-related disease. Notably, to our knowledge, this is the first report of a patient with MGORS7 showing an encouraging clinical response to PEG-rhGH therapy. This preliminary clinical observation suggests that GH therapy may have potential benefits for growth retardation associated with this disorder. Although this is a single-case study, continued follow-up and studies involving larger patient cohorts are warranted to further evaluate the long-term efficacy and safety of GH therapy.

## Data Availability

The datasets presented in this article are not readily available because of ethical and privacy restrictions. Requests to access the datasets should be directed to the corresponding authors.
