# Supplementary material for: Identification of a novel and a recurrent CDC45 variant in a Chinese family with Meier-Gorlin syndrome 7 and a literature review
Source: Front Genet. 2026 Aug 24;17:1893476. doi: 10.3389/fgene.2026.1893476 (PMC13546744; doi:10.3389/fgene.2026.1893476)
Supplement: Supplementary file 1 [file Table1.pdf]

Supplement table 1. Quality control metrics of whole-genome sequencing

	data size	20× coverage	average cover (×)
**Patient**	139.1G	0.9357	44.22
**Father**	145.4G	0.9417	45.93
**Mother**	172.8G	0.9596	54.74
